# Difference in Cerebral and Hepatic Oxygenation in Response to Ultrafiltration in a Hemodialysis Patient With Congestive Heart Failure

**DOI:** 10.7759/cureus.13023

**Published:** 2021-01-30

**Authors:** Yuko Mutsuyoshi, Kiyonori Ito, Susumu Ookawara, Takayuki Uchida, Yoshiyuki Morishita

**Affiliations:** 1 Division of Nephrology, First Department of Integrated Medicine, Jichi Medical University Saitama Medical Center, Saitama, JPN; 2 Department of Clinical Engineering, Jichi Medical University Saitama Medical Center, Saitama, JPN

**Keywords:** cerebral oxygenation, congestive heart failure, hemodialysis, hepatic oxygenation, regional oxygen saturation

## Abstract

Near-infrared spectroscopy has been used to measure regional oxygen saturation (rSO_2_), and intradialytic tissue rSO_2_ measurements have been playing an important role in evaluating changes in tissue oxygenation in various clinical settings of hemodialysis (HD) therapy.However, few reports have described changes in hepatic oxygenation associated with body fluid management in overhydrated HD patients. We herein report an HD patient with congestive heart failure (CHF) that had improved systemic and tissue oxygenation, including in the brain and liver, during HD with ultrafiltration. A 73-year-old man undergoing HD was admitted to our hospital with CHF. After admission, HD with ultrafiltration was performed to adequately manage his body fluid excess. Because of deterioration of systemic oxygenation on admission, we monitored his percutaneous arterial oxygen saturation (SpO_2_) using a pulse oximeter and regional oxygen saturation (rSO_2_) in the brain and liver using an INVOS 5100c oxygen saturation monitor during HD. At HD initiation, his cerebral and hepatic rSO_2_ levels were relatively low, at 43.2% and 34.1%, respectively, in addition to the SpO_2_ of 88%. During HD with ultrafiltration, systemic oxygenation evaluated by SpO_2_ and tissue oxygenation by cerebral and hepatic rSO_2_ improved. Interestingly, the hepatic rSO_2_ ratio, defined as the ratio of rSO_2_ values at *t *(min) during HD and the initial rSO_2_ value before HD, increased larger than the cerebral rSO_2_ ratio during HD. After the adjustment of body fluid condition under the maintained SpO_2_ values, we confirmed the hepatic and cerebral SO_2_ ratio again during HD, and these two values changed nearly in the same manner. Throughout our experience, in this case, we confirmed a remarkable increase in hepatic rSO_2_ ratio relative to cerebral rSO_2_ ratio under a CHF status during HD, and these differences disappeared after the adjustment of the body fluid status.

## Introduction

Cardiovascular disease is known as one of the major complications in chronic kidney disease (CKD) patients, including those on hemodialysis (HD). Its prevalence has been affected by multiple factors, including body fluid excess, hypertension, anemia, and mineral bone disorder [1]. In addition, heart failure was reportedly the most important cause of death in patients on HD [2]. Therefore, adequate body fluid management is necessary to prevent the onset of cardiovascular disease, including congestive heart failure (CHF), and to improve patient prognosis [3,4].

Near-infrared spectroscopy has been used to measure regional oxygen saturation (rSO2), and intradialytic tissue rSO2 measurements, including those of the brain and liver, have been playing an important role in evaluating changes in tissue oxygenation in various clinical settings of HD therapy. Thus far, it has been reported that cerebral rSO2 is low at the onset of acute CHF and improves throughout the management of body fluid excess during HD with ultrafiltration [5], although cerebral and hepatic rSO2 were maintained during HD without intradialytic hypotension [6,7]. However, few reports have described changes in hepatic oxygenation associated with body fluid management in overhydrated HD patients. In this paper, we report an HD patient with CHF that had improved systemic and tissue oxygenation, including in the brain and liver, during HD. Furthermore, the relative increase in hepatic rSO2 might be larger than that in cerebral rSO2 in response to the improvement of body fluid status induced by ultrafiltration during HD.

## Case presentation

A 73-year-old man undergoing HD was referred to our hospital because of chest discomfort and dyspnea. His medical history included hypertension, diabetes mellitus, and myocardial infarction. At the time of referral, his body weight increased by 4.6 kg, which was nearly equivalent to 10% of his body weight, from the end of the last HD therapy two days ago. Furthermore, his chest radiograph showed perihilar vascular engorgement, and he was diagnosed with acute CHF due to excess body fluid. After admission, HD with ultrafiltration was performed to adequately manage his body fluid excess. Table 1 shows the vital signs, laboratory findings, and systemic oxygenation status before HD.

**Table 1:** Vital signs, laboratory findings, and oxygenation status at first and second measurement of systemic and tissue oxygenation before hemodialysis

	First measurement (on admission)	Second measurement (4thhospital day)
Oxygen inhalation status	NPPV with FiO_2 _of 0.6	Room air
Hemodialysis- associated parameters		
Blood Pressure (mmHg)	104/54	121/65
Pulse Rate (beats/min)	117	84
Body Weight (Kg)	50.9	48.6
Ultrafiltration (L/session)	1.3	2.2
Laboratory findings		
Hemoglobin (g/dL)	12.1	10.2
Total protein (g/dL)	6.3	5.9
Serum albumin (g/dL)	3.6	3.3
Blood urea nitrogen (mg/dL)	72	40
Serum creatinine (mg/dL)	10.9	9.2
Parameters in systemic and tissue oxygenation		
SpO_2_ (%)	88	97
Cerebral rSO_2_ (%)	43.2	46.7
Hepatic rSO_2_ (%)	34.1	36.7

**Figure 1:**
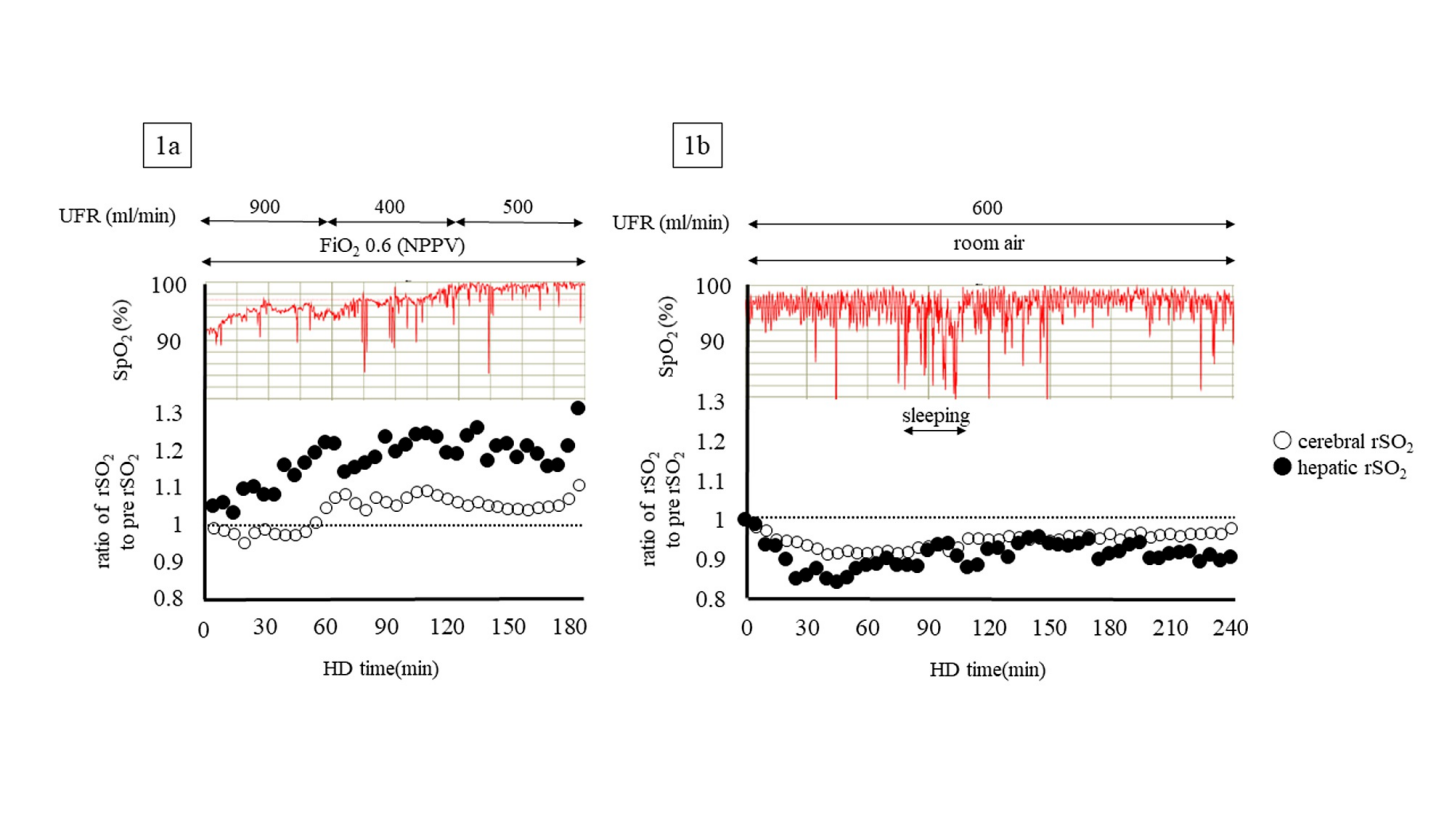
Changes in SpO2, and cerebral and hepatic rSO2 during HD 1a: under a CHF status with NPPV, 1b: under stable HD in room air; SpO2, saturation of percutaneous oxygen; rSO2, regional saturation of oxygen; HD, hemodialysis; CHF, congestive heart failure; NPPV, noninvasive positive-pressure ventilation; UFR, ultrafiltration; FiO2, fraction of inspiratory oxygen

This patient provided informed consent before evaluation, and we monitored his percutaneous arterial oxygen saturation (SpO2) using a pulse oximeter (PULSOX-Me300; Teijin Pharma, Tokyo, Japan) and rSO2 in the brain and liver using an INVOS 5100c oxygen saturation monitor during HD. At HD initiation, cerebral and hepatic rSO2 levels were 43.2% and 34.1%, respectively, in addition to the SpO2 of 88%, even with the use of noninvasive positive pressure ventilation with oxygen administration. Throughout this HD procedure, systemic oxygenation evaluated by SpO2 and tissue oxygenation by cerebral and hepatic rSO2 improved (Figure 1a), and respiratory management using noninvasive positive pressure ventilation was able to be completed at the end of HD. Interestingly, the hepatic rSO2 ratio, which was defined as the ratio of rSO2 values at t (min) during HD and the initial rSO2 value before HD, increased larger than the cerebral rSO2 ratio during HD (Figure 1a). On the 4th hospital day without his respiratory symptoms under the maintained SpO2 values, we again confirmed the hepatic and cerebral SO2 ratio during HD. Before HD without oxygen inhalation, cerebral and hepatic rSO2 levels were 46.7 % and 36.7%, respectively (Table 1). Furthermore, values in cerebral and hepatic rSO2 were changed nearly in the same manner throughout the HD procedure (Figure 1b), in contrast to changes in those during HD with an overhydrated status.

## Discussion

The progression of CKD has been associated with several major complications. Cardiovascular disease, including heart failure, is a major complication induced by excess sodium with fluid retention; its prevalence and severity also increase from early stage CKD to end-stage renal disease [1]. Indeed, heart failure was the most common cause of death (23.5%) in all-cause deaths in dialysis patients [2]. Therefore, in the field of HD therapy, it is important to adequately manage patients’ body fluid status to prevent the prevalence of heart failure [3,4]. However, the causes of heart failure are known to be multifactorial [1]; therefore, it may be hard to completely prevent the onset of CHF in the clinical setting of HD therapy. Thus far, reports regarding tissue oxygenation, including the brain and liver, in patients with CHF and on HD have been limited. A review of the data and findings in previous reports of acute phase CHF and changes in tissue oxygenation are summarized in Table 2 [5,8,9].

**Table 2:** Changes in tissue oxygenation throughout the treatment of congestive heart failure

Authors	Year	Dialysis therapy	Number of patients	Monitoring area	rSO_2_ (%) at onset of CHF	rSO_2_ (%) after CHF treatment	Main results about tissue oxygenation during CHF treatment	reference
Madsen PL, et al.	2000	No	9	Brain	34	50	Cerebral oxygen saturation is low at onset of CHF and increases with the well-being of the patient by the successful treatment.	[8]
Hogan CJ, et al.	2011	No	19 (non-adverse outcome)	Hypothenar region of the palm	61.2	66.3	Tissue oxygenation increased during treatment in CHF patients without future adverse outcomes. However, it was unchanged for those who later died or were readmitted. Lack of tissue oxygenation improvement maybe associated with higher rates of death and readmission.	[9]
			33 (adverse outcome*)		57.6	60.2		
Minato S, et al.	2019	HD	1		NPPV with FiO_2 _of 0.4	Room air	Cerebral oxygenation deteriorated with the CHF status but was improved by body-fluid management during HD.	[5]
				Brain	34	45		

Only three reports were found, which discussed the association between them. Two out of the three reports did not include patients with dialysis therapy [8,9] and only one case report was for an HD patient [5]. Tissue oxygenation was measured at the forehead [5,8] and hypothenar [9] and improved after CHF treatment in all reports. Particularly, ultrafiltration during HD had a positive effect on cerebral oxygenation [5] and lack of tissue oxygenation improvement might be associated with higher rates of death and readmission [9].

In HD patients without intradialytic hypotension, cerebral and hepatic rSO2 at HD initiation were 46.5 ± 1.3% and 52.4 ± 1.7%, respectively [7]. Furthermore, these rSO2 values were relatively constant despite ultrafiltration and cerebral rSO2 values were significantly lower than hepatic rSO2, which might be a specific feature of tissue oxygenation in HD patients without excess body fluid [7]. Recently, in a patient with acute CHF on HD, cerebral rSO2 reportedly increased during HD with ultrafiltration according to the systemic oxygenation increase [5]. This increase in cerebral rSO2 might have originated from the improvement of cerebral microcirculation associated with body fluid adjustment, in addition to the improvement of systemic oxygenation. Therefore, the increase in cerebral rSO2 in response to the improvement in body fluid status, in this case, might be consistent with a previous report [5]. Furthermore, hepatic rSO2 was 34.1%, which was relatively low compared to that in 43.2% of cerebral rSO2 in this case. However, the hepatic rSO2 ratio rapidly increased by ultrafiltration, and the degree of increase in hepatic rSO2 ratio was larger than that in cerebral rSO2 ratio during HD. Therefore, different from the relationship between cerebral and hepatic oxygenation without excess body fluid in HD patients [7], the deterioration in hepatic oxygenation may be larger than that in cerebral oxygenation under overhydrated status. In addition, hepatic oxygenation may be likely to improve compared to cerebral oxygenation according to the body fluid improvement induced by ultrafiltration. In this case, there might be a possibility to increase hepatic rSO2 beyond cerebral rSO2 in response to the further improvement in body fluid status, although hepatic rSO2 value was still low compared to that in cerebral rSO2 at second measurement after the improved CHF.

After his respiratory symptoms disappeared on the fourth hospital day, SpO2 dramatically improved at HD initiation even without oxygen inhalation. However, cerebral and hepatic rSO2 less improved compared to the SpO2 improvements. Previous studies reported no significant association between cerebral rSO2 and SpO2 in HD patients [10-12]. Therefore, the improvement of systemic oxygenation in the central circulation might be different from changes in tissue oxygenation in the microcirculation. That is, the improvement of microcirculation in tissues may be delayed compared to that of central circulation throughout the body fluid adjustment in HD patients. However, fluid status in the microcirculation could not be accurately evaluated and we cannot directly comment on these associations. Therefore, further studies are needed to investigate the mechanism of changes in systemic and tissue oxygenation in response to ultrafiltration in acute CHF.

## Conclusions

We confirmed a remarkable increase in hepatic rSO2 ratio relative to cerebral rSO2 ratio under a CHF status during HD, and these differences disappeared after the adjustment of the body fluid status.
